# Association between different types of smoking and first sexual behavior in adolescents: Survey data from South Korea

**DOI:** 10.18332/tid/200197

**Published:** 2025-02-14

**Authors:** Yunyun Wu, Wenbin Du

**Affiliations:** 1 Research Institute of Social Development, Southwestern University of Finance and Economics, Chengdu, China

**Keywords:** adolescent, gender differences, tobacco use, South Korea, initiation of sexual behavior

## Abstract

**INTRODUCTION:**

Adolescent deviant behaviors can severely hinder their healthy development. This study explores the relationship between tobacco consumption and sexual behavior in Korean adolescents.

**METHODS:**

This is a secondary dataset analysis of the pooled KYRBS cross-sectional studies from 2018 to 2022. Sexual behavior generally refers to physical contact or interactions related to sex. In this study, sexual behavior primarily refers to intercourse. A logit model was applied to examine the relationship between different types of tobacco use and the initiation of sexual behavior. Furthermore, data from the KYRBS 2018–2021 were used to analyze the time-related patterns in the connection between tobacco use and the first occurrence of sexual behavior among adolescents, with a focus on the moderating role of gender.

**RESULTS:**

Tobacco use among adolescents was positively associated with the occurrence of sexual behavior. After adjusting for other variables, adolescents who use ordinary cigarettes (AOR=7.85; 95% CI: 7.49–8.23), nicotine e-cigarettes (AOR=8.21; 95% CI: 7.80–8.64), or combustible e-cigarettes (AOR=9.86; 95% CI: 9.26–10.49) were more likely to engage in sexual behavior than non-users. Further research indicates that the earlier an individual reported beginning smoking tobacco, the earlier their first sexual encounter occurs, the correlation coefficient is 0.526 (p<0.01). Additionally, in the moderation analysis, the male group weakened the trend in the time to first tobacco use and first sexual behavior.

**CONCLUSIONS:**

The use of different types of tobacco among Korean adolescents is strongly associated with sexual behavior. Adolescents are starting to engage in sexual activity at a younger age, especially males. Thus, providing reproductive health education to adolescent smokers could enhance their sexual decision-making. Furthermore, implementing tobacco cessation and sexual education for adolescents necessitates the development of gender-specific strategies to meet the distinct needs of males and females.

## INTRODUCTION

Adolescence is a critical stage for an individual’s physiological, psychological, and socio-behavioral development, with the initiation of sexual behavior often considered an important indicator of health-related behaviors^1^. Earlier initiation of sexual behavior is a significant indicator of risks in adolescents, including unplanned pregnancies, abortions, and sexually transmitted diseases (STDs)^2^. Between 2013 and 2022 in South Korea, the rate of sexual experience among male adolescents increased from 7.4% to 7.6%, while among female adolescents, it rose from 3.1% to 4.7%^3^. The 14th Korean Youth Risk Behavior Web-based Survey (2018) reported that 48.3% of male adolescents and 42.1% of female adolescents used contraceptives during sexual encounters, with the average age of first sexual experience being 13.6 years^3^. The initiation of sexual behavior is associated with adolescents’ reproductive health and closely linked to their psychological development, social adaptability, and behavioral patterns^4^. Early sexual behavior is often accompanied by adverse consequences such as teenage pregnancies, sexually transmitted diseases (STDs), gender-based violence, and psychological health issues^5^. Among South Korean adolescents, the incidence of first sexual behavior has been rising annually, largely influenced by the internet and social media platforms, which have contributed to a younger age of early sexual behavior and a higher rate. Another study shows a declining trend in the average age of first sexual experience among South Korean adolescents in recent years, with some individuals in specific regions experiencing their first sexual behavior before the age of 16 years^6^. In Korean society, which is characterized by a relatively conservative outlook, the premature initiation of sexual activity is regarded as a societal taboo^7^. Although the incidence of sexual behavior in South Korea is lower than in Western countries, the emerging trend of earlier sexual activity is of significant research importance^7^. Therefore, exploring the factors influencing the initiation of sexual behavior among South Korean adolescents is essential for developing early intervention and preventive strategies for this group, which has become a significant issue in the field of public health^8^.

The literature examining the determinants of initial sexual behavior among adolescents predominantly focuses on identifying pertinent risk factors. These determinants can be categorized into multiple factors, including physiological, psychological, familial, societal, and environmental aspects^9^. Among these, smoking behavior is widely acknowledged as a significant factor with potential implications within the behavioral patterns of adolescents^10^. Previous research has shown that adolescent smoking is related to various adverse health behaviors, including alcohol consumption and drug abuse^11^. Crucially, smoking may also be associated with the early onset of sexual behavior. Specifically, an earlier age of smoking initiation is potentially related to earlier sexual activity^12^. Early sexual behavior in adolescents can increase the risks of unintended pregnancies and sexually transmitted infections^13,14^. Human papillomavirus (HPV), which has been established as the causative agent in the development of cervical cancer, can be more effectively prevented by delaying the age at first sexual intercourse and reducing the number of sexual partners. Meanwhile, smoking cessation can significantly decrease the incidence of cervical cancer in young women^15^. Additionally, the risk of contracting HIV is higher among adolescents who initiate sexual activity at an early age^16^. A relevance study between early smoking, risk-taking behaviors, and reproductive health found that early smokers were more likely to experience adolescent pregnancies, miscarriages, and at least one sexually transmitted infection than those who smoked later. This suggests that the age at which smoking commenced may predict future reproductive health^17^. However, the emergence of electronic cigarettes and heated tobacco products has added complexity to the issue, extending the impact of smoking behavior beyond traditional cigarette use. In recent years, the smoking rate among Korean adolescents has risen, particularly due to the increase in electronic cigarette use. Younger individuals perceive these products as ‘low-risk’ or ‘fashionable’ alternatives, posing a new health threat^18^. There remains a lack of systematic empirical research to determine whether there is a significant correlation between smoking behavior and initial sexual behavior among adolescents, particularly with respect to the differential use of cigarettes, electronic cigarettes, and heated tobacco products.

Adolescence is a period characterized by the emergence of numerous issues, such as alcohol consumption, smoking, and drug abuse, and it is also a time when interest in the opposite sex intensifies. The health consequences following sexual behavior during adolescence include an increased risk of sexually transmitted infections, psychosocial impacts stemming from unwanted sexual relationships, and other adverse health behaviors^19^.

This study aims to facilitate smoking cessation guidance from the perspective of sexual behavior. Therefore, it investigates the following: 1) the association between tobacco use types and the occurrence of sexual behavior; and 2) the relationship between the timing of first tobacco use and first sexual behavior, as well as the moderating effect of gender.

## METHODS

### Data sources and sample selection

**Figure 1:**
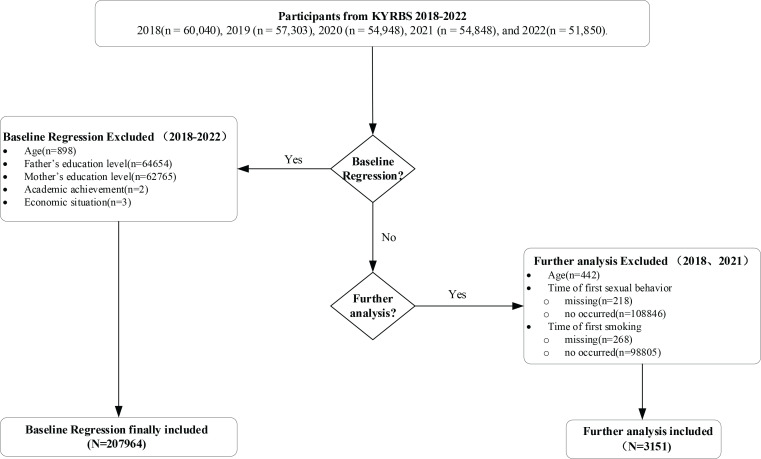
Flowchart of participants selection, South Korea (2018–2022)

This study is a secondary analysis of the Korea Youth Risk Behavior Web-based Survey (KYRBS), an anonymous online survey conducted annually by the Korea Disease Control and Prevention Agency (KDCA) since 2005. The KYRBS provides national data on risk behaviors among Korean adolescents and uses a two-stage stratified cluster sampling design. The KYRBS data are available for free download on the official KYRBS website (http://yhs.cdc.go.kr). The sample is stratified into 117 strata based on 39 regional clusters and three levels of education (middle schools, general high schools, and specialized high schools). Participants are equally distributed across all grade levels in 800 middle and high schools. The study samples were derived from the KYRBS conducted in 2018 (n=60040), 2019 (n=57303), 2020 (n=54948), 2021 (n=54848), and 2022 (n=51850). Data from KYRBS 2018 to KYRBS 2022 were compiled into a mixed cross-sectional dataset. This study used Stata 17.0 software for the analysis. All statistical tests were two-tailed and the significance levels are as follows: (*p<0.1, **p<0.05, ***p<0.01). In studies examining early sexual behavior among Korean minors, 207964 samples were included in the regression analysis after excluding missing values. Furthermore, since information on the timing of first sexual behavior was only collected in KYRBS 2018 and 2021, we selected data from these two years for further analysis, with a final sample size of 3151 after excluding missing values (Figure 1).

### Outcome variable

This study’s dependent variable is the sexual behavior of Korean adolescents, focusing on both the occurrence of sexual activity and the timing of first sexual intercourse. The sexual behavior of adolescents was evaluated through the following questions: ‘Have you had sexual relations?’ and ‘When was the first time you had sex?’. For the first question, responses were recoded such that affirmative answers were coded as 1, and negative answers were coded as 0. Participants who gave an affirmative response to the first question proceeded to answer the second question. The responses to the second question were treated as a continuous variable, with values ranging from 1 to 13, corresponding to ‘before entering school’ to ‘the third year of high school’. Higher values indicate a later timing of initial sexual intercourse.

### Predictive variables

*Smoking or not*

The independent variable in this study is adolescent smoking behavior, specifically focusing on three types of tobacco products: general cigarettes (cigarettes), liquid e-cigarettes with nicotine and cigarette-type electronic cigarettes (such as iCos, Glo, Lil, etc.). Three binary variables were created based on the following questionnaire items: ‘Have you ever used conventional cigarettes (tobacco cigarettes)?’, ‘Have you ever used liquid e-cigarettes with nicotine so far?’, and ‘Have you ever used cigarette-style electronic cigarettes (heated tobacco products, such as iCos, Glo, Lil)?’. Responses were categorized as ‘yes’ or ‘no’, with ‘yes’ assigned a value of 1 and ‘no’ a value of 0.

*The time of initial cigarette use*

To analyze the timing of adolescents’ first cigarette use, we excluded respondents who indicated they had never smoked. For the remaining participants, their reported age at first cigarette use was transformed into a continuous variable ranging from 1 to 13, representing different stages or ages of smoking initiation.

### Controlled variables

We have introduced a set of covariates that may influence adolescent sexual behavior. These include gender, age, family socioeconomic status (rated on a 1–5 scale, with higher scores indicating better socioeconomic status), academic performance (scored on a scale of 1–5, with higher scores indicating better performance), grade level, fathers’ education level, mothers’ education level, educational stage, school type, school level and city size.

### Statistical analysis

First, descriptive analyses were conducted to summarize the characteristics of all variables. Concurrently, the annual proportions of adolescents using different types of cigarettes was calculated. These types were categorized into four groups: non-users, single-type users, dual-type users, and triple-type users. Second, since sexual activity is a dummy variable and the use of an Ordinary Least Squares (OLS) model could lead to heteroskedasticity, we employed a logit model for regression analysis in this study and provided adjusted odds ratios (AORs) and 95% confidence intervals (95% CIs). Specifically, Models 1–3 investigate the association between tobacco use and sexual behavior, whereas Model 4 incorporates all three tobacco types into the regression analysis. The OLS model was used in further analysis to examine the association between the timing of initial cigarette use and sexual initiation. Additionally, the moderating effect of gender was introduced. In the moderation effect analysis, an interaction term between gender and the timing of first tobacco use was included. In the baseline regression analysis, control variables included gender, age, economic situation, academic achievement, grade, parental education level, educational stage, school type, and city scale. In the further moderation effect analysis, control variables included economic situation, academic achievement, grade, educational stage, school type, and city scale. All analyses control for time and city fixed effects Third, Kaplan-Meier (KM) survival estimates, which combine event outcomes (sexual activity occurrence) with time to event (timing of sexual activity), provide more reliable visual results. We shall present a graph where the x-axis represents survival time (adolescent age), while the y-axis represents the survival rate (proportion of individuals who have engaged in sexual activity in the total sample). A value of 1 on the y-axis indicates that no individuals in the sample have engaged in sexual activity. The line segments in the graph represent the proportion of individuals who have engaged in sexual activity within each age group. In comparison to the Kaplan-Meier (KM) survival curve, the Cox regression analysis estimates the risk ratio for event time data, considering the effects of grade, academic achievement, educational stage, school type, and city scale.

## RESULTS

### Descriptive statistical analysis

**Figure 2:**
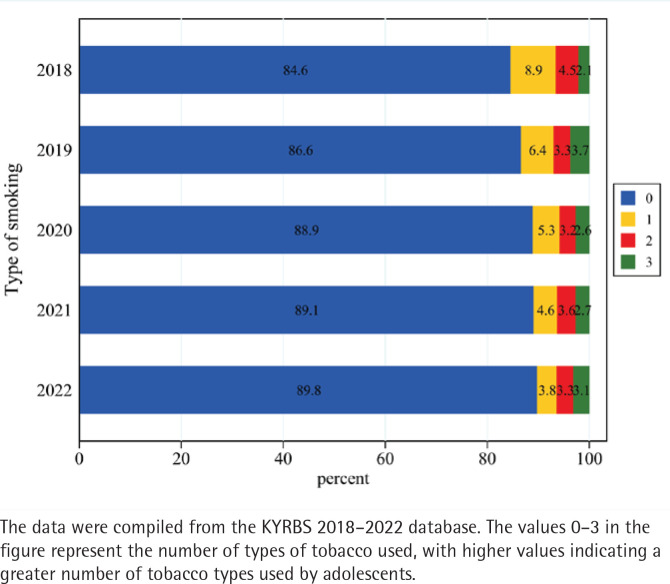
The trend in the number of types of tobacco used by adolescents, South Korea (2018–2022) (N=207964)

Table 1 provides descriptive statistics for the key variables. The proportion of male students was 48.4%, while female students accounted for 52.6%, resulting in a nearly equal gender ratio. The average age of the adolescents was approximately 14.9 years. Most adolescents perceived their family’s economic status as moderate. Students who maintained upper middle academic performance constituted the largest proportion, with 86.5% achieving grades at or above the intermediate level. The majority of parents had completed college education. In terms of school types, mixed-gender schools were the most prevalent, followed by girls’ schools and boys’ schools, which account for 17% and 15%, respectively. Regarding city size, students from small- and medium-sized cities represented the largest group, followed by those from large cities, while those from county areas are the least numerous. The mean average prevalence (proportion within the sample) of sexual behavior among Korean adolescents was 0.044. The rate of adolescent sexual activity increased from 4.69% in 2018 to 4.93% in 2022. Among the three types of tobacco products, conventional cigarettes had the highest usage rate, averaging 10% of adolescents. Additionally, Figure 2 reveals a declining trend in adolescents who use only one type of cigarette, with the majority having experience with multiple types of tobacco products.

**Table 1:** Descriptive statistics for all variables, by smoking or not, South Korea (2018–2022) (N=207964)

*Variables*	*Overall* *(N=207964)*			*Smoking* *(N1=185029)*	*No smoking* *(N2=22935)*	*Mean test*	
	*Proportion*	*n*	*Mean*	*Mean 1*	*Mean 2*	*Diff*	*p*
**Sexual behavior**			0.044	0.023	0.214	-0.19	0.000***
Yes	0.044	9240					
No	0.956	198724					
**Gender**			0.484	0.459	0.688	-0.228	0.000***
Male	0.484	100736					
Female	0.526	107228					
**Age** (years) (range: 12–18 years)			14.94	14.827	15.866	-1.039	0.000***
**Family economic situation** (1–5)			3.404	3.416	3.306	0.110	0.000***
Disadvantaged	0.016	3335					
Low-income	0.093	19334					
Middle-income	0.474	98611					
High-income	0.305	63359					
Affluent	0.112	23325					
**Academic achievement** (1–5)			3.143	3.197	2.705	0.492	0.000***
Poor	0.087	18037					
Below average	0.216	44817					
Average	0.301	62566					
Good	0.262	54525					
Excellent	0.135	28019					
**Grade** (1–6)			3.313	3.198	4.248	-1.051	0.000***
1st junior high school	0.193	40143					
2nd junior high school	0.183	38136					
3rd junior high school	0.176	36593					
1st high school	0.155	32207					
2nd high school	0.150	31251					
3rd high school	0.142	29634					
**Father’s education level**			2.936	2.952	2.801	0.151	0.000***
Junior middle school	0.014	2973					
Senior middle school	0.226	47081					
University	0.569	118280					
Other	0.191	39630					
**Mother’s education level**			2.889	2.904	2.765	0.139	0.000***
Junior middle school	0.011	2382					
Senior middle school	0.263	54749					
University	0.550	114475					
Other	0.175	36358					
**Educational stage**			1.448	1.417	1.691	-0.273	0.000***
Middle school	0.552	114872					
High school	0.448	93092					
**School type**			1.980	1.962	2.127	-0.165	0.000***
Girls’ school	0.175	36308					
Co-educational	0.671	139456					
Boys’ school	0.155	32200					
**City scale**			1.636	1.632	1.666	-0.034	0.000***
Big city	0.438	91159					
Small- and medium-sized city	0.488	101414					
County district	0.074	15391					

*** p<0.01.

### Regression results

Table 2 presents an analysis of the association between the use of different types of cigarettes and sexual behavior, with adjustments made for potential confounding factors. The findings of Models 1–3 indicate that, under constant other conditions, smoking, whether conventional cigarettes or e-cigarettes, was significantly associated with the occurrence of sexual behavior among minors. Specifically, there was a positive association between smoking behavior and sexual activity, exhibiting a convergent trend. Individuals who used ordinary cigarettes were more likely to engage in sexual behavior than those who do not (AOR=7.85; 95% CI: 7.49–8.23). Adolescents who use liquid e-cigarettes with nicotine were more likely to engage in sexual behavior than those who do not (AOR=8.21; 95% CI: 7.80–8.64). When controlling for all other variables, adolescents who use combustible electronic cigarettes were more likely to engage in sexual behavior compared to those who do not (AOR=9.86; 95% CI: 9.26–10.49). In Model 4, the regression analysis included all three types of cigarettes, and the results remained statistically significant. However, the coefficients were smaller compared to those from individual analyses; specifically, ordinary cigarette (AOR=4.20; 95% CI: 3.94–4.48), liquid e-cigarettes with nicotine (AOR=2.02; 95% CI: 1.87–2.18), and cigarette type e-cigarette (AOR=2.17; 95% CI: 2.01–2.34).

**Table 2:** Logit regression results on the relationship between adolescent tobacco use types and the occurrence of sexual behavior, South Korea (2018–2022) (N=207964)

*Variables*	*Sexual behavior* *AOR (95% CI)*			
	*Model 1*	*Model 2*	*Model 3*	*Model 4*
Ordinary cigarette	7.85 (7.49–8.23)***			4.20 (3.94–4.48)***
Liquid e-cigarettes with nicotine		8.21 (7.80–8.64)***		2.02 (1.87–2.18)***
Cigarette type e-cigarette			9.86 (9.26–10.49)***	2.17 (2.01–2.34)***
Gender	1.26 (1.19–1.34)***	1.30 (1.22–1.37)***	1.46 (1.38–1.54)***	1.17 (1.10–1.24)***
Age (years)	1.15 (1.09–1.20)***	1.15 (1.10–1.21)***	1.15 (1.10–1.21)***	1.14 (1.09–1.19)***
Economic situation	1.09 (1.06–1.12)***	1.08 (1.05–1.10)***	1.07 (1.05–1.10)***	1.07 (1.04–1.10)***
Academic achievement	0.920 (0.902–0.939)***	0.903 (0.885–0.921)***	0.879 (0.862–0.897)***	0.941 (0.923–0.960)***
Grade	1.18 (1.12–1.25)***	1.22 (1.16–1.29)***	1.24 (1.17–1.30)***	1.19 (1.13–1.25)***
Father’s education level	0.933 (0.898–0.970)***	0.923 (0.888–0.960)***	0.912 (0.877–0.948)***	0.938 (0.902–0.976)***
Mother’s education level	0.928 (0.892–0.965)***	0.920 (0.884–0.956)***	0.907 (0.872–0.943)***	0.928 (0.892–0.966)***
Educational stage	1.12 (1.02–1.24)**	1.07 (0.973–1.18)	1.08 (0.979–1.18)	1.07 (0.970–1.18)
School type	1.07 (1.02–1.12)***	1.07 (1.02–1.12)***	1.08 (1.03–1.13)***	1.07 (1.02–1.12)***
City scale	1.14 (1.04–1.24)***	1.14 (1.04–1.25)***	1.10 (1.01–1.20)**	1.12 (1.03–1.23)**
Year FE	Yes	Yes	Yes	Yes
City FE	Yes	Yes	Yes	Yes
pseudo R^2^	0.170	0.156	0.138	0.187

AOR: adjusted odds ratio. Models 1 to 3 sequentially display the relationships between various types of tobacco use (ordinary cigarette, liquid e-cigarettes with nicotine, and cigarette type e-cigarette) and the occurrence of sexual behavior. Model 4 includes all three types of tobacco in the analysis. In each model, the covariates are presented, and both time and city fixed effects are included.

** p<0.05,

*** p<0.01.

Among the control variables, compared to females, male adolescents exhibit a significant positive correlation with the occurrence of sexual behavior (p<0.01). Adolescent males were significantly more likely to engage in sexual behavior than females (AOR=1.17; 95% CI: 1.10–1.24). The probability of adolescents engaging in sexual behavior increased with age. For each unit increase in age, the likelihood of engaging in sexual behavior increased by 0.14% (AOR=1.14; 95% CI: 1.09–1.19). A better family economic status was associated with a higher probability of sexual behavior (AOR=1.07; 95% CI: 1.04–1.10). Higher grade level was associated with an increased probability of sexual behavior (AOR=1.19; 95% CI: 1.13–1.25). Academic performance (AOR=0.941; 95% CI: 0.923–0.960), father’s education level (AOR=0.938; 95% CI: 0.902–0.976), and mother’s education level (AOR=0.928; 95% CI: 0.892–0.966) were protective factors against engaging in sexual behavior. Students in co-educational and boys’ schools were more likely to engage in sexual behavior than those in girls’ schools. School type with a higher proportion of males was associated with a higher probability of sexual behavior (AOR=1.07; 95% CI: 1.02–1.12). Larger city size was associated with a higher probability of engaging in sexual behavior (AOR=1.12; 95% CI: 1.03–1.23) (Tables 2 and 3).

**Table 3:** Regression results of the number of tobacco types used and the occurrence of sexual activity, South Korea (2018–2022) (N=207964)

	*Sexual behavior*			
	*OR*	*SE*	*Z*	*95% CI*
**Number of tobacco types**				
1	4.911***	0.161	48.4	4.61–5.24
2	8.681***	0.303	62.0	8.11–9.30
3	17.93***	0.642	81.0	16.7–19.2
**Gender**	1.16***	0.034	4.87	1.09–1.22
**Age** (years)	1.14***	0.027	5.55	1.09–1.19
**Economic situation**	1.07***	0.014	5.18	1.04–1.10
**Academic achievement**	0.943***	0.010	-5.82	0.924–0.962
**Grade**	1.19***	0.032	6.30	1.13–1.25
**Father’s education level**	0.939***	0.019	-3.15	0.902–0.976
**Mother’s education level**	0.931***	0.019	-3.54	0.895–0.969
**Educational stage**	1.07	0.052	1.37	0.972–1.18
**School type**	1.068***	0.026	2.76	1.02–1.12
**City scale**	1.12**	0.052	2.53	1.03–1.23
**pseudo R^2^**	0.189			

AOR: adjust odds ratio. SE: standard error.

** p<0.05,

*** p<0.01.

**Figure 3:**
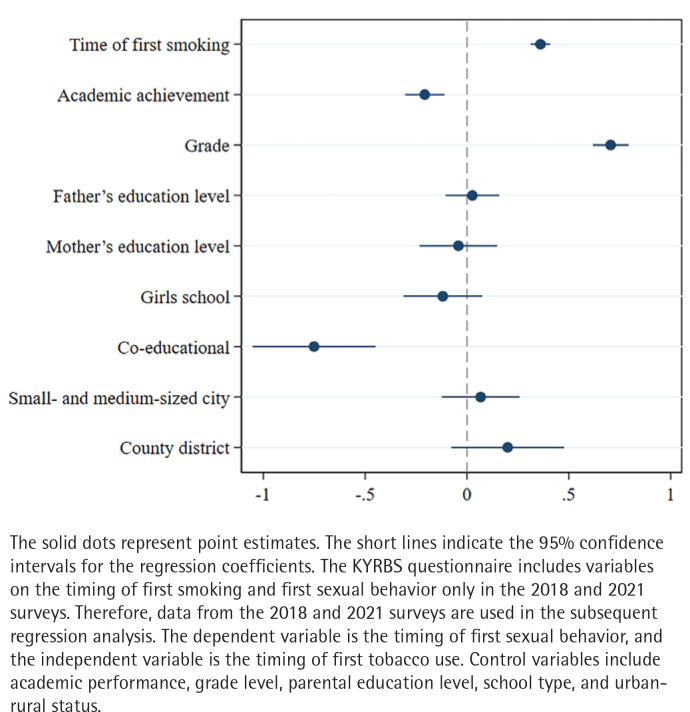
The visualization of the regression results for first-time smoking and first-time sexual activity, South Korea (2018–2021) (N=3151)

Further analysis examines the relationship between smoking and sexual behavior in the time dimension. Table 4 Model 1 shows that: under constant conditions, for every unit increase in the time of first use of tobacco, the time to first sexual intercourse increases by 0.526 indicating that earlier tobacco use is possibly associated with earlier sexual behavior. The visualization results in Figure 3 further validate the findings.

**Table 4:** Moderating effect of gender, South Korea (2018–2021) (N=3151)

	*Time of first sexual behavior (OLS)*	
	*Model 1*	*Model 2*
Time of first smoking	0.526 (0.026)***	0.591 (0.039)***
Gender (Female as reference group)	0.247(0.111)**	1.07 (0.452)**
Gender × Time of first smoking		-0.100 (0.05)**
Grade	0.604 (0.063)***	0.602 (0.063)***
Academic achievement	-0.287 (0.040)***	-0.286 (0.039)***
Educational stage	0.073 (0.205)	0.071 (0.205)
**School type** (Girls school as reference group)		
Co-educational	-0.341 (0.122)***	-0.284 (0.120)**
Boys school	-0.837 (0.172)***	-0.785 (0.171)***
City scale	0.035 (0.176)	0.022 (0.177)
Constant	3.08 (0.472)***	2.51 (0.546)***
Year FE	Yes	Yes
City FE	Yes	Yes
N	3151	3151
R^2^	0.347	0.349
adj. R^2^	0.342	0.343

Standard errors in parentheses.

** p<0.05,

*** p<0.01.

### The analysis of moderating effects

This analysis explores gender differences in the relationship between smoking and the timing of first sexual behavior. Figure 4A uses survival analysis, revealing that males initiate sexual behavior earlier than females. The log-rank test was significant [χ^2^(1)=430.09, Pr >χ^2^=0.0000]. Figure 4B further validates this result through the use of Cox proportional hazards regression. Table 4, Model 2 introduces gender as a moderating variable and creates an interaction term between gender and the timing of first tobacco use. The results show a positive correlation between the timing of first tobacco use and first sexual behavior (p<0.01), with a coefficient of 0.591. Gender was also positively associated with the timing of first sexual behavior. However, the coefficient of the interaction term is -0.100, suggesting that males reduce the positive association between the timing of first tobacco use and first sexual behavior. The visualization in Figure 5 clearly illustrates that gender significantly inhibits this relationship, with the effect being stronger for males.

**Figure 4A:**
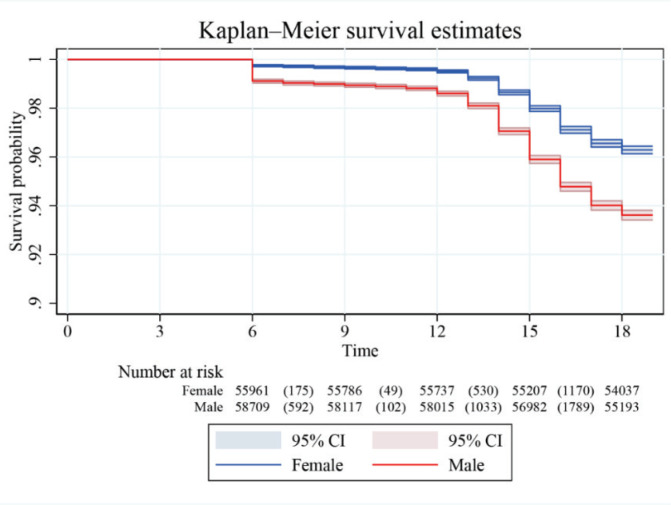
Kaplan-Meier (KM) survival estimates of initial sexual behavior by gender, South Korea (2018–2021)

**Figure 4B:**
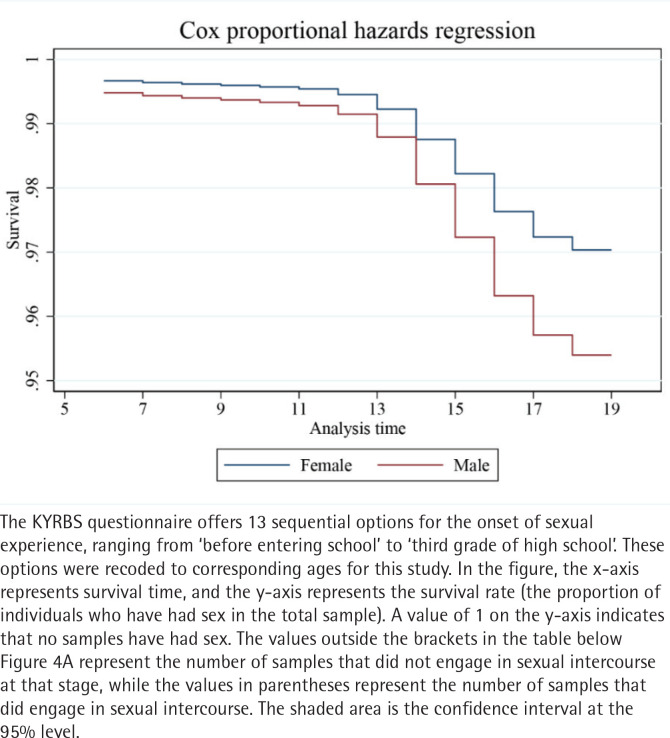
Cox proportional hazards regression of initial sexual behavior by gender, South Korea (2018–2021)

**Figure 5:**
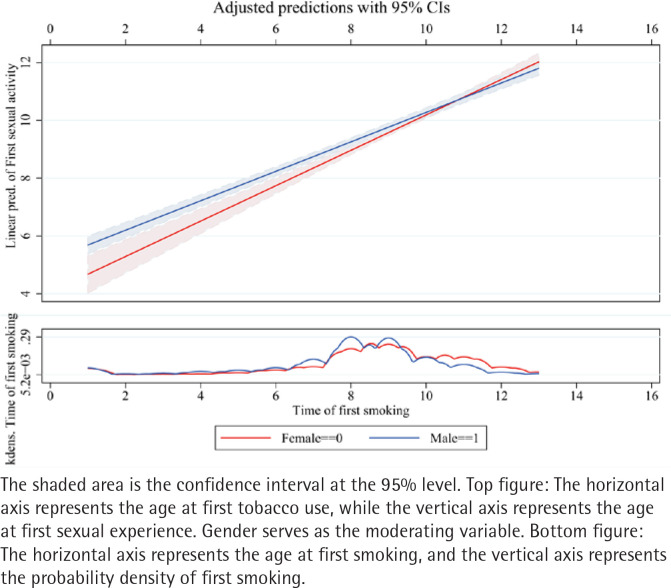
Moderating effect of gender, South Korea (2018–2021) (N=3151)

## DISCUSSION

Tobacco use is a significant public health issue that leads to numerous health consequences, including respiratory diseases and various cancers^20^. Adolescence represents a crucial period for the formation of healthy behavioral patterns, and both tobacco use and premature sexual activity can have long-term negative impacts on adolescents’ physical and mental well-being. Thus, it is crucial to conduct comprehensive research on these behaviors. In this study, we first employed a logit model to identify a positive correlation between smoking and sexual behavior among Korean adolescents. The act of smoking is widely regarded as a dangerous practice, which is closely associated with adolescents’ traits such as thrill-seeking and a sense of adventure^21^. In social interactions, adolescents may be profoundly influenced by their peers or subcultural groups, perceiving smoking as a fashionable and avantgarde symbol. They may attempt to reinforce their status and sense of identity among their peers through this behavior^22^. These factors collectively contribute to adolescents engaging in sexual behavior at an earlier age. Furthermore, relevant research indicates that during adolescence, the brain regions associated with decision-making, such as the prefrontal cortex, are not fully developed, resulting in their weaker abilities in impulse control and prediction of long-term consequences^23^. Smoking is a behavior marked by low self-control and high impulsivity, which may also influence sexual behavior through similar deficits in impulse control^24^. Therefore, smoking and early sexual behavior may reflect a common psychological and physiological trait: impulsivity and a lack of effective self-regulation.

We also found that adolescents who use e-cigarettes are more inclined to participate in sexual behavior compared to those who use conventional cigarettes. On the one hand, e-cigarettes, with their novel and fashionable designs, exert a strong appeal on adolescent populations. On the other hand, this is associated with the ‘gateway effect’ of e-cigarettes^25^. Research indicates that e-cigarette users are more likely than non-users to begin using conventional cigarettes^26^. This outcome indicates that the use of e-cigarettes may result in smoking behavior among adolescents. Meanwhile, the media is a potential factor contributing to adolescents’ exposure to secondhand smoke (SHS)^27^. Exposure to e-cigarette advertisements increases the likelihood of future e-cigarette use and is also linked to traditional cigarette use. Among current smokers, those who smoke more frequently are more inclined to utilize electronic cigarettes for the purpose of quitting smoking^28^. Furthermore, electronic cigarettes are less subject to venue restrictions and do not require the concealment of odors when used, which undoubtedly enhances adolescents’ acceptance of and frequency of using electronic cigarettes^29^.

Despite the misconception among adolescents that e-cigarettes may pose a lower health risk compared to conventional tobacco cigarettes, they actually remain a nicotine-addictive substance^30^. In particular, young people face a greater risk of addiction when using electronic cigarettes^31^. In October 2018, the World Health Organization (WHO), during the Eighth Session of the Conference of the Parties to the Framework Convention on Tobacco Control (FCTC), reached an agreement calling for the application of the same policies and regulations to electronic cigarettes and heated tobacco products as those applied to traditional tobacco. As a member state of the FCTC, South Korea has gradually intensified its regulation of electronic cigarettes and heated tobacco products. For example, e-liquid containing nicotine extracted from tobacco stems or roots was officially classified as tobacco in 2021^32^. So far, numerous novel products, such as e-cigarettes containing synthetic nicotine, have not been fully incorporated into tobacco regulations, creating regulatory gaps. Additionally, adolescents still have access to promotional e-cigarette advertisements through social media, websites, and blogs^33^. These advertisements may reinforce the misconception among adolescents that e-cigarettes are relatively ‘harmless’^34^. This cognitive bias causes adolescents to underestimate the health risks of e-cigarettes, increasing both their acceptance and frequency of use^34^. This perception of ‘harmlessness’ may indicate adolescents’ reduced ability to assess other risks, especially when making risk-taking decisions related to sexual behavior, where similar cognitive biases may be present^24^. In other words, adolescents may develop a mindset of ‘I can control or evade the consequences’ when using electronic cigarettes, and this psychological bias may extend to their decision-making processes involving sexual behavior. Consequently, the utilization of e-cigarettes among teenagers is not only a warning sign of health risks but may also affect their perception and behavioral patterns towards other potential risks. Therefore, strengthening the regulation of electronic cigarettes, particularly in guiding and educating adolescent populations, is of paramount importance.

This study used Kaplan-Meier (KM) survival estimates to reveal a significant positive correlation between smoking initiation and sexual behavior initiation, with gender playing a key moderating role. This can be explained by the innate tendency of males to take risks in many domains^35^. In many cultures, males tend to engage in sexual behavior earlier, as they may be more inclined to exhibit dominant behavior within their peer group, which is closely related to ‘masculine traits’ such as the pursuit of excitement, risk-taking, and expectations of gender roles^21^. Simultaneously, males’ sexual behavior may also be influenced by peer pressure, particularly in male-dominated social circles where early sexual behavior may be viewed as a sign of maturity or social acceptance^36^. Continued attention to adolescent sexual behavior is essential. These findings not only enhance our understanding of adolescents’ health behaviors but also provide a scientific foundation for the development of targeted health education and intervention strategies.

### Limitations

Although this study controls for time and city fixed effects, it relies on mixed cross-sectional data, which cannot fully capture unobserved individual-level variations over time or cannot establish causal relationships. Second, self-reported assessments may introduce reporting biases. Third, residual confounding factors may remain because not all potential confounders (such as peer influences and major family events) were controlled for in the analysis. Unmeasured or inadequately controlled variables, such as mental health status or previous exposure to risk behaviors, may still affect the observed relationships. Fourth, the generalizability of the results may be limited. The study’s target population consists of Korean adolescents. Additionally, tobacco policies, cultural norms, and sexual attitudes vary across countries, which could influence the study’s applicability to other settings. Therefore, the specific context of this study may not be applicable to other settings or populations, particularly those with different cultural or policy environments.

## CONCLUSIONS

This research explored the connection between adolescent tobacco use and sexual behavior, revealing a statistically significant link between tobacco use and early sexual activity. Specifically, a significant positive association was observed between the age at first smoking and the age of first sexual experience. Furthermore, when gender was analyzed as a moderating variable, results showed that boys who started smoking earlier were more likely to engage in earlier sexual activity.

## Data Availability

The data that support the findings of this study are available from the Korea Youth Risk Behavior Web-based Survey (KYRBS) (http://yhs.cdc.go.kr)
